# *MTHFR* C677T, A1298C and *MS* A2756G Gene Polymorphisms and Male Infertility Risk in a Chinese Population: A Meta-Analysis

**DOI:** 10.1371/journal.pone.0169789

**Published:** 2017-01-12

**Authors:** Zhengju Ren, Pengwei Ren, Bo Yang, Kun Fang, Shangqing Ren, Jian Liao, Shengzhuo Liu, Liangren Liu, Zhufeng Peng, Qiang Dong

**Affiliations:** 1 Department of Urology, Institute of Urology, West China Hospital, Sichuan University, Chengdu, Sichuan, China; 2 Department of Evidence-Based Medicine and Clinical Epidemiology, West China Hospital, Sichuan University, Chengdu, China; Central South University, CHINA

## Abstract

**Background:**

Methylenetetrahydrofolate reductase gene (*MTHFR* C677T and A1298C) and methionine synthase gene (*MS* A2756G) polymorphisms have shown an association with male infertility risk in several ethnic populations. Although several studies have evaluated these associations in Chinese populations, their small sample sizes and inconsistent outcomes have prevented strong conclusions. Therefore, the present meta-analysis was performed with published studies to evaluate the associations of the three single nucleotide polymorphisms (SNPs) and male infertility in a Chinese population.

**Methods:**

We conducted a search of PubMed, Embase, Web of Science, Chinese National Knowledge Infrastructure (CNKI), China biology medical literature (CBM), VIP, and Chinese literature (Wan Fang) databases up to May 31, 2016. Odds ratios (ORs) and 95% confidence intervals (95%CIs) were used to assess the strength of associations with a random-effect model or a fixed-effect model based on the heterogeneity analysis results. Sensitivity analysis was used to confirm the reliability and stability of the meta-analysis.

**Results:**

A total of nine studies, including 1,713 cases and 1,104 controls, were included in the meta-analysis. The pooled results indicated that the *MTHFR* C667T polymorphism was significantly associated with increased risk of male infertility in the Chinese population in the allele model (T vs. C: OR = 1.47, 95%CI = 1.32–1.63), the dominant model (TT + CT vs. CC: OR = 1.51, 95%CI = 1.30–1.77), the additive model (TT vs. CC: OR = 2.08, 95%CI = 1.68–2.58) and the recessive model (TT vs. CT+CC: OR = 1.58, 95%CI = 1.31–1.90), whereas the *MTHFR* A1298C and *MS* A2756G polymorphisms were not risk factors. There was no significant heterogeneity in any genotype contrasts among the studies. The sensitivity analysis indicated that the results of this meta-analysis were relatively stable.

**Conclusion:**

This study suggests that the *MTHFR* C667T polymorphism may contribute to the genetic susceptibility to male infertility in the Chinese population, whereas *MTHFR* A1298C and *MS* A2756G polymorphisms may be unrelated to male infertility. Studies with larger sample sizes and representative population-based cases and well-matched controls are needed to validate our results.

## Introduction

Infertility is defined as the failure of a couple to achieve pregnancy after one year of unprotected, regular sexual intercourse, which affects approximately 15% of all couples attempting to conceive a child[1, 2]. In addition to environmental and lifestyle risk factors, genetic causes, such as chromosomal aberrations and single gene mutations, also play important roles in male infertility. Among the well-known genes that cause male infertility, such as *FSHR*[3], *AR*[4], *PRM1*[5], and *GST*[6], the folate-related enzyme genes are those most often involved.

Folate plays an important role in DNA synthesis, RNA synthesis, methylation reactions, and protein synthesis, which contribute to the maintenance of genome integrity[7, 8]. Several single-nucleotide polymorphisms (SNPs) of folate metabolism-related genes have been identified, including methylenetetrahydrofolate reductase (MTHFR; 607093) gene polymorphisms (*MTHFR* C677T, rs1801133 and *MTHFR* A1298C, rs1801131), a methionine synthase (MS; 156570) gene polymorphism (*MS* A2756G, rs1805087, also known as *MTR* A2756G), and a methionine synthase reductase (MTRR; 602568) gene polymorphism (*MTRR* A66G, rs1801394). These SNPs can affect the activity, stability, and level of folate metabolism-related enzymes, which may affect folate metabolism and DNA synthesis[9]. Folate metabolism disorder may lead to sperm DNA damage and spermatogenic failure[10].

To date, several studies have explored the associations between these SNPs and male infertility risk; however, their results are conflicting. As a result, several meta-analyses addressing these associations have been performed. Three recent meta-analyses consistently showed that the *MTHFR* C677T polymorphism was associated with a significantly increased male infertility risk in the Asian and overall populations but not the Caucasian population[11–13]. Two recent meta-analyses both showed that the *MS* A2756G polymorphism may be a genetic risk factor for idiopathic male infertility[13, 14]. Moreover, two recent meta-analyses were performed to examine the association between *MTHFR* A1298C and the risk of male infertility, the results were inconsistent[11, 13]. In the Chinese population, several studies have examined the associations between folate-related enzyme gene polymorphisms and the risk of male infertility; however, the results are inconclusive. Because the majority of relevant studies in the Chinese population were published in local Chinese journals, most international readers cannot access and/or read them. In addition, the recent meta-analyses do not include all relevant studies of Chinese populations[11–15]. Therefore, to evaluate the relationships between each of the three SNPs and male infertility risk within the Chinese population, we performed a meta-analysis including the most recent data in the literature. To our knowledge, this is the first meta-analysis performed on this topic in the Chinese population.

## Methods

### Search strategy

**Fig 1:**
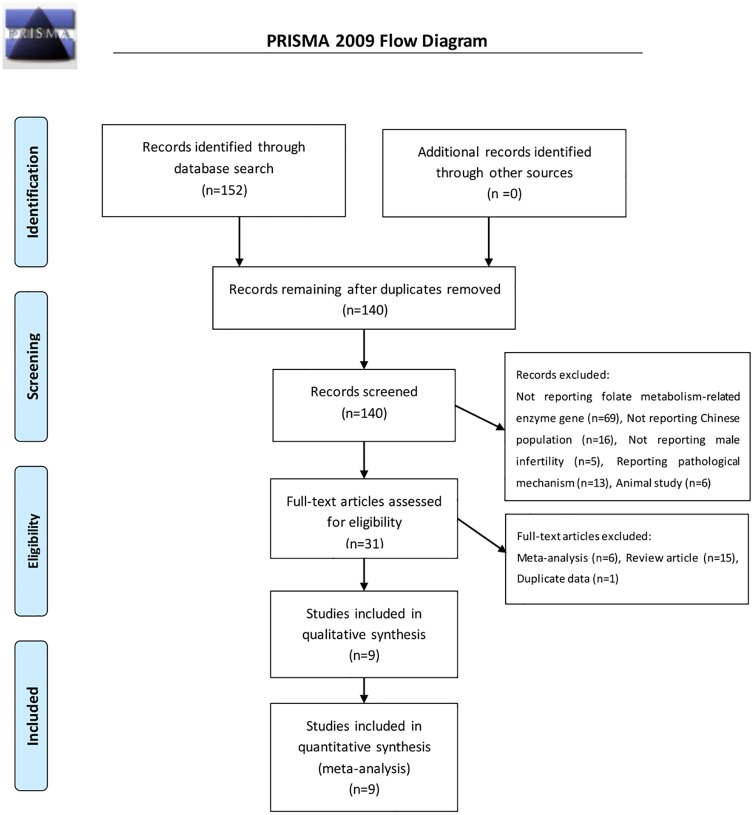
Flowchart of the study selection procedure.

Two authors independently conducted a systematic literature search of the PubMed, Embase, Web of Science, Chinese National Knowledge Infrastructure (CNKI), China biology medical literature (CBM), VIP, and Chinese literature (Wan Fang) databases up to May 31, 2016. Search terms were as follows: “MTHFR or Methylenetetrahydrofolate reductase”, “MTR, MS or methionine synthase”, “SNP, polymorphism, mutation, or variant”, “male infertility”. In addition, the references of reviews and retrieved articles were reviewed to identify other eligible studies that were missed by the search. The search was limited to human subjects. The search strategy flowchart is shown in Fig 1.

### Inclusion and exclusion criteria

Only those studies meeting the following inclusive selection criteria were eligible: 1) The full text of the article was available. 2) The study was a case—control study evaluating at least one of the three SNPs. 3) The genotype distributions were available for both cases and controls. 4) There were no duplicate data. For studies that considered partially or fully duplicate data and that were by the same authors, we selected the study with the most subjects. 5) The published language was English or Chinese. 6) The study was of a Chinese population. 7) Genotypic distributions were available for the estimation of odds ratios (ORs) and 95% confidence intervals (CIs). Studies were excluded if any of the following criteria existed: 1) The study did not explore the association between any of the three SNPs and male infertility risk. 2) The article was an animal study, review article, meta-analysis, conference abstract or editorial article.

### Quality assessment

The Newcastle-Ottawa Scale (NOS)[16] was used to assess the quality of the included studies. The NOS contains eight items for both cohort and case—control studies. The scale assesses the quality of case-control studies based on three areas: selection, comparability, and exposure. A “star” rating system is used to judge the methodological quality. Selection has a maximum of 4 stars, comparability has a maximum of 2 stars, and exposure has a maximum of 3 stars. The total scores ranged from 0 stars (worst) to 9 stars (best), and the quality of each study was graded as low (0–3), moderate (4–6), or high (7–9). Discrepant opinions were resolved by discussion and consensus.

### Data extraction strategy

Two authors extracted the relevant data independently in compliance with the inclusion criteria. Extracted data were entered into a collection form and checked by a third author. Disagreement was solved by discussion and consensus. Data on the following variables for each study were extracted: 1) first author’s name, year of publication, region, and genotyping method; 2) sample sizes of the case and control groups; 3) genotype and allele frequencies; and 4) results of the Hardy—Weinberg equilibrium test.

### Statistical analysis

The strength of the relationships between the *MTHFR* gene polymorphisms and male infertility risk were assessed using ORs and corresponding 95% CIs. The pooled ORs were calculated for the allele comparison model, dominant model, recessive model, and codominant model. The heterogeneity assumption was tested using the Chi-square-based Q test. Heterogeneity was considered significant at p<0.10, and *I*^*2*^ values of 25%, 50% and 75% corresponded to low, medium and high levels of heterogeneity, respectively. The significance of the pooled ORs were determined by the Z-test, and P<0.05 was considered statistically significant. The statistical analysis was performed with Reviewer Manager 5.3 and STATA 12.0. Potential publication bias was estimated using funnel plots and the Egger regression test. Sensitivity analysis was performed to evaluate the stability of the results. The pooled ORs were estimated by excluding one study each time to evaluate the influence of individual studies.

## Results

### Study characteristics

A total of 152 results were retrieved from the search of the PubMed, Embase, Web of Science, Chinese National Knowledge Infrastructure (CNKI), China biology medical literature (CBM), VIP, and Chinese literature (Wan Fang) databases. Three studies were excluded because they were meta-analyses as determined from reading the title and abstract. An additional two publications contained duplicate data and were published by the same author; the one with the most subjects was included in the present analysis. Nine case-control studies considering 1,713 cases and 1,104 controls met the inclusion criteria[17–25](Fig 1). Of these, all 9 studies addressed the *MTHFR* C667T polymorphism; 3 studies addressed *MTHFR* A1298C polymorphism, and 3 studies addressed the *MS* A2756G polymorphism. The year of publication ranged from 2007 to 2015. The Hardy-Weinberg test (HWE) was performed on all of the included studies, and HWE of the *MTHFR* C667T polymorphism was violated in one study[25]. The characteristics of each of the included studies are shown in Table 1. The quality of studies based on the NOS score is presented in Table 2.

**Table 1:** Characteristics of the studies included in the meta-analysis and their genotype distributions of the *MTHFR* C677T, *MTHFR* A1298C and *MS* A2756G gene polymorphisms.

	Study	Region	Genotyping method	case	control	case					control					
						CC	CT	TT	C	T	CC	CT	TT	C	T	HWE
*MTHFR*C667T	Ni et al.2015	Zhejiang	SNaPshot	296	204	117	135	44	369	223	84	94	26	262	146	0.970
	Li et al.2015	Sichuan	Sequencing	162	120	61	77	24	199	125	48	54	18	150	90	0.661
	Li et al.2014	Beijing	PCR-RFLP	82	133	14	36	32	64	100	36	61	36	133	133	0.340
	Pei et al.2013	Henan	PCR	190	90	39	138	113	216	364	24	47	19	95	85	0.651
	Liu et al.2012	Shenzhen	PCR	75	72	27	38	10	92	58	40	28	4	108	36	0.753
	Qiu et al.2011	Ningxia	PCR	271	180	75	112	84	262	280	63	85	32	211	149	0.720
	Sun et al.2007	Jilin	PCR	182	53	27	86	69	140	224	15	28	10	58	48	0.630
	Yang et al.2010	Anhui	PCR-RFLP	131	293	34	55	42	123	139	98	142	53	338	248	<0.05
	A et al.2007	Sichuan	PCR-RFLP	355	252	130	160	65	420	290	128	95	29	351	153	0.085
						AA	AC	CC	A	C	AA	AC	CC	A	C	
*MTHFR*A1298C	Ni et al.2015	Zhejiang	SNaPshot	296	204	181	106	9	468	124	137	62	5	336	72	0.515
	Li et al.2015	Sichuan	Sequencing	162	120	101	54	7	256	68	80	38	2	198	42	0.290
	Li et al.2014	Beijing	PCR-RFLP	82	133	49	29	4	127	37	88	36	9	212	54	0.060
						AA	AG	GG	A	G	AA	AG	GG	A	G	
*MS* A2756G	Li et al.2015	Sichuan	Sequencing	162	120	124	35	3	283	41	101	17	2	219	21	0.220
	Ni et al.2015	Zhejiang	SNaPshot	296	204	245	47	4	537	55	163	37	4	363	45	0.280
	Liu et al.2012	Shenzhen	PCR	75	72	60	14	1	134	16	61	11	0	133	11	0.480

**Table 2:** Quality assessment for all of the included studies.

First author	Publishing year	Selection	Comparability	Exposure	Total
Ni	2015	★★★	NA	★★	5
Li	2015	★★★	★	★★	6
Li	2014	★★★	★★	★★	7
Pei	2013	★★★	★	★★	6
Liu	2012	★★★	★★	★★	7
Qiu	2011	★★	★	★★	5
Sun	2007	★★★	★	★★	6
Yang	2010	★★★	NA	★★	5
A	2007	★★★	★	★★	6

### Association of the *MTHFR* C667T polymorphism with male infertility

**Fig 2:**
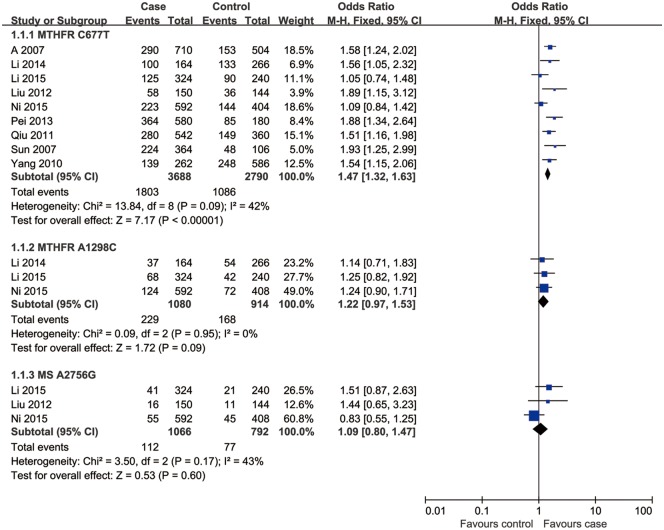
Forest plot of the studies assessing the association between *MTHFR* C677T, *MTHFR* A1298C and *MS* A2756G polymorphisms and male infertility. (allelic model: (a) T vs. C, (b) C vs. A, (c) G vs. A).

**Fig 3:**
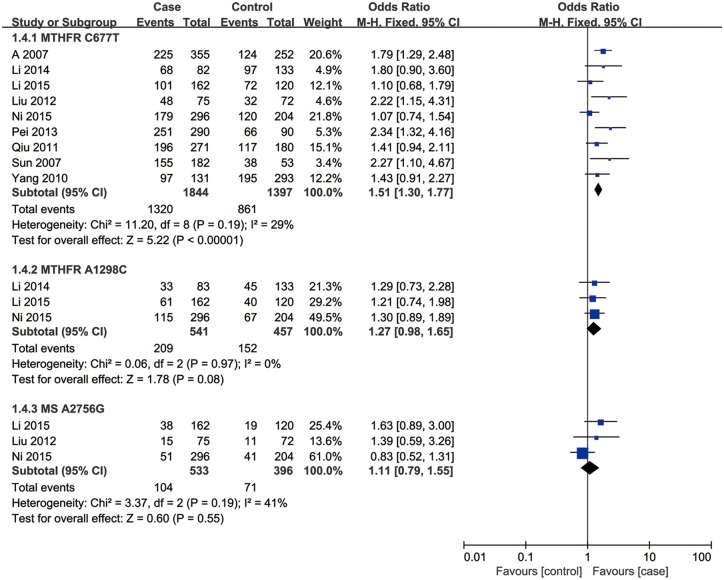
Forest plot of the studies assessing the association between *MTHFR* C677T, *MTHFR* A1298C and *MS* A2756G polymorphisms and male infertility. (dominant model: (a) TT+CT vs. CC, (b) CC+AC vs. AA, (c) GG +AG vs. AA).

**Fig 4:**
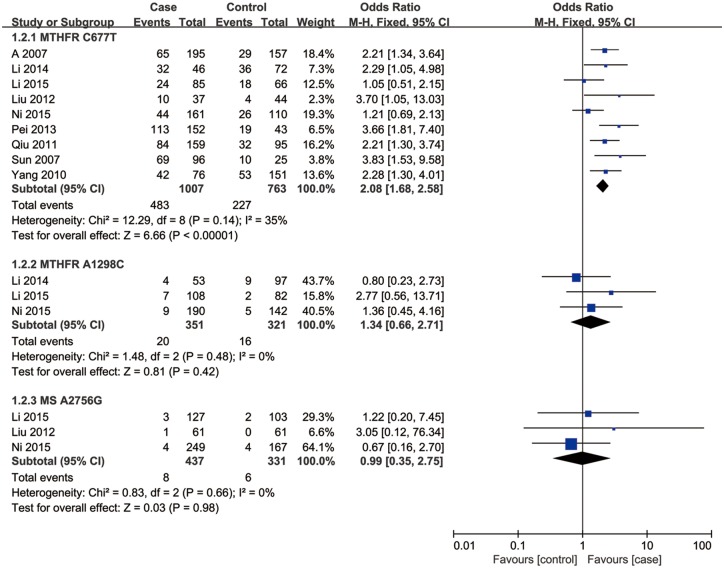
Forest plot of the studies assessing the association between *MTHFR* C677T, *MTHFR* A1298C and *MS* A2756G polymorphisms and male infertility. (additive model: (a) TT vs. CC, (b) CC vs. AA, (c) GG vs. AA).

**Fig 5:**
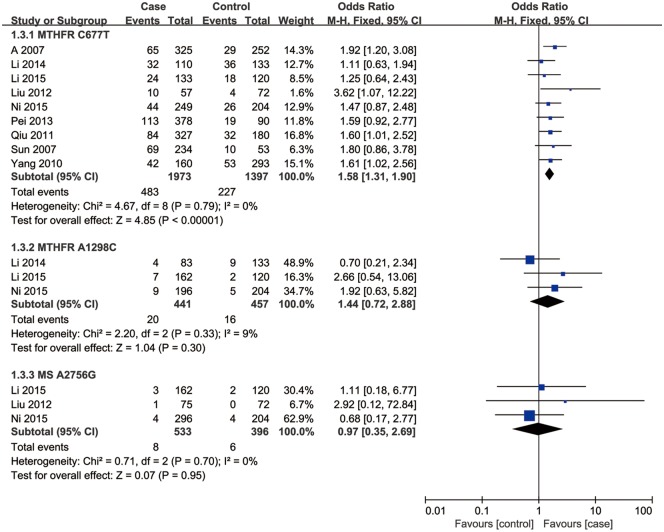
Forest plot of the studies assessing the association between *MTHFR* C677T, *MTHFR* A1298C and *MS* A2756G polymorphisms and male infertility. (recessive model: (a) TT vs. CC+CT, (b) CC vs. AA+AC, (c) GG vs. AA+AG).

Nine studies involving a total of 2,817 individuals evaluated the influence of the *MTHFR* C667T polymorphism on the risk of male infertility. Figs 2–5 shows the meta-analysis results for the allele model (T/C), dominant model (TT+CT vs. CC), additive model (TT/CC) and recessive model (TT vs. CC+CT), for which the *I*^2^ value, representing the among-study heterogeneity, was 42%, 29%, 35%, and 0%, respectively. Thus, fixed-effects models were applied. Overall, the results revealed a significant association between the *MTHFR* C677T polymorphism and Chinese male infertility risk (T vs. C: OR = 1.47, 95%CI = 1.32–1.63; TT + CT vs. CC: OR = 1.51, 95%CI = 1.30–1.77; TT vs. CC: OR = 2.08, 95%CI = 1.68–2.58; TT vs. CT+CC: OR = 1.58, 95%CI = 1.31–1.90) (Figs 2–5).

### Association of *MTHFR* A1298C and *MS* A2756G polymorphisms with male infertility

Three studies including a total of 898 individuals evaluated the influence of the *MTHFR* A1298C polymorphism on the risk of male infertility. There was no significant heterogeneity in any genotype contrasts among the studies, and fixed-effects models were applied. Overall, the results revealed no association between the *MTHFR* A1298C polymorphism and Chinese male infertility risk in the allele model (C vs. A: OR = 1.22, 95%CI = 0.97–1.53, *I*^2^ = 0), dominant model (CC + AC vs. AA: OR = 1.27, 95%CI = 0.98–1.65, *I*^2^ = 0), additive model (CC vs. AA: OR = 1.34, 95%CI = 0.66–2.71, *I*^2^ = 0) or recessive model (CC vs. AC+AA: OR = 1.44, 95%CI = 0.72–2.88, *I*^2^ = 9) (Figs 2–5).

Three studies, including a total of 929 individuals, evaluated the influence of the *MS* A2756G polymorphism on the risk of male infertility. There was no significant heterogeneity in any genotype contrasts among the studies, and fixed-effects models were applied. Overall, the results revealed no association between the *MS* A2756G polymorphism and Chinese male infertility risk without heterogeneity in the additive model (GG vs. AA: OR = 0.99, 95%CI = 0.35–2.75, *I*^2^ = 0) or recessive model (GG vs. AG+AA: OR = 0.97, 95%CI = 0.35–2.69, *I*^2^ = 0) and no association between the polymorphism and infertility risk with low heterogeneity in the allele model (G vs. A: OR = 1.09, 95%CI = 0.80–1.47, *I*^2^ = 43) or dominant model (GG + AG vs. AA: OR = 1.11, 95%CI = 0.79–1.55, *I*^2^ = 41) (Figs 2–5).

### Sensitivity and publication bias

**Fig 6:**
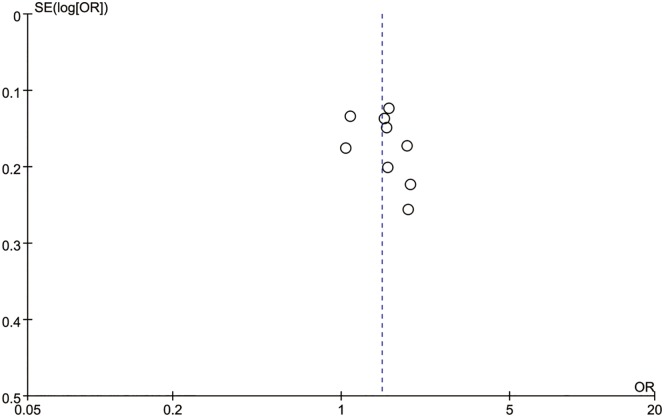
Funnel plot for the *MTHFR* C677T polymorphism and male infertility risk in the Chinese population. (allelic model: T vs. C).

**Fig 7:**
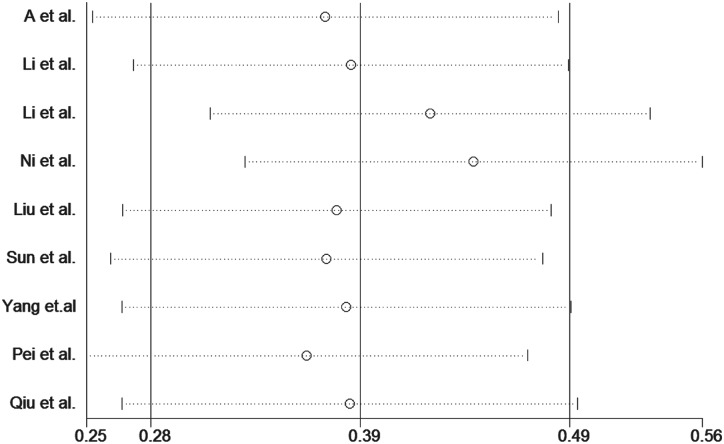
Sensitivity analysis diagram for each study used to assess the relative risk estimates for the *MTHFR* C677T polymorphism and male infertility in all of the included studies. (allelic model: T vs. C).

**Fig 8:**
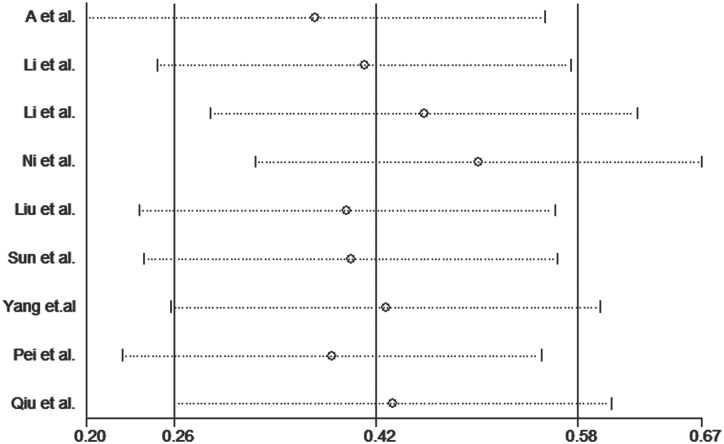
Sensitivity analysis diagram for each study used to assess the relative risk estimates for the *MTHFR* C677T polymorphism and male infertility in all of the included studies. (dominant model: TT + TC vs. CC).

**Fig 9:**
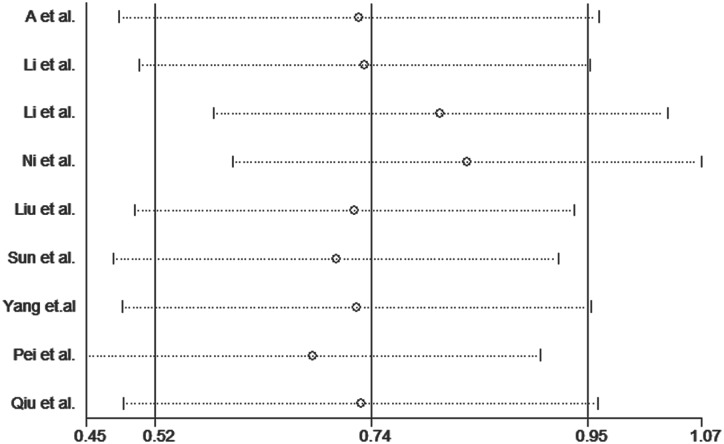
Sensitivity analysis diagram for each study used to assess the relative risk estimates for the *MTHFR* C677T polymorphism and male infertility in all of the included studies. (additive model: TT vs. CC).

**Fig 10:**
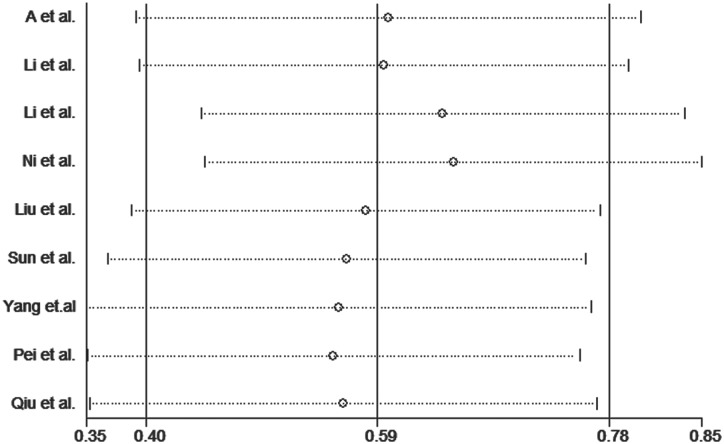
Sensitivity analysis diagram for each study used to assess the relative risk estimates for the *MTHFR* C677T polymorphism and male infertility in all of the included studies. (recessive model: TT vs. TC + CC).

Publication bias was assessed for the *MTHFR* C667T polymorphism by funnel plots, Begg’s test and Egger’s test under all contrast models. The shape of the funnel plot did not indicate any evidence of obvious asymmetry in any contrast model for the *MTHFR* C667T polymorphism (Fig 6). In addition, Egger’s linear regression analysis suggested no evidence of publication bias (P = 0.99 for an allelic contrast model, P = 0.91 for a codominant model, P = 0.77 for a recessive model, and P = 0.51 for a dominant model) (Table 3). We did not produce funnel plots for the other two single nucleotide polymorphisms (SNPs) due to the limited number of studies on *MTHFR* A1298C and *MS* A2756G polymorphisms. The sensitivity analyses were conducted to calculate the pooled ORs by omitting one study each time. The results showed that no individual study influenced the overall pooled ORs (Figs 7–10), indicating that the results of this meta-analysis are relatively stable.

**Table 3:** Publication bias test for the *MTHFR* C677T polymorphism.

Comparisons	Egger test			Begg test
	Coefficient	P value	95% CI	P value
T vs. C	-0.04	0.99	-7.51 7.42	0.47
TT vs. CC	-0.09	0.91	-1.85 1.68	0.12
TT vs. CC+CT	-0.05	0.77	-0.44 0.34	0.75
CT+TT vs. CC	-1.8	0.51	-7.98 4.38	0.47

## Discussion

Folate-mediated one-carbon metabolism is essential for many reactions in human cells, such as DNA methylation, DNA repair and DNA synthesis[26, 27]. Abnormal folate metabolism has been proposed as a factor in male infertility. Methylenetetrahydrofolate reductase (MTHFR) and methionine synthase (MS) are the key enzymes implicated in the folate metabolic pathways and are crucial for DNA methylation and spermatogenesis. The single nucleotide polymorphisms (SNPs) of these folate-related enzymes gene can impair folate absorption or disturb the balance between folate derivatives by impacting the activity, stability, or level of the corresponding enzymes. The mechanisms of pathogenesis may involve changes of enzyme structure and mRNA properties that are due to these folate-related enzymes gene polymorphisms[28]. Recent studies have revealed that folate-related enzyme gene polymorphisms were associated with an increased risk of male infertility, particularly in the case of *MTHFR* gene polymorphisms[1, 29, 30]. Although many studies have reported associations between *MTHFR* and *MS* gene polymorphisms and male infertility risk[14, 31], no meta-analysis to date has comprehensively evaluated the relationships of *MTHFR* and *MS* gene polymorphisms with male infertility risk in the Chinese population. Hence, we performed such a meta-analysis.

In the present study, a meta-analysis was conducted of nine case-control studies to evaluate the association between three folate-related enzyme gene polymorphisms and male infertility in the Chinese population. Overall, we did not find the variant genotypes of the *MTHFR* A1298C and *MS* A2756G polymorphisms to be associated with male infertility risk. However, a significant association between the *MTHFR* C667T polymorphism and male infertility was detected (OR: 1.47, allelic genetic model; OR: 1.58, recessive genetic model; OR: 1.51, dominant genetic model; OR: 2.08, codominant genetic model). The results are consistent with recent meta-analysis studies that suggest a moderate to strong association between *MTHFR* C677T and male infertility, especially in Asian populations[11–13, 32].

Ni et al. reported that the *MTHFR* C667T polymorphism was not a risk factor for male infertility risk in a Chinese population, in contrast to the conclusions of a previous study. Similarly, Li et al. found no evidence an association of this polymorphism with male infertility risk. This difference among studies may be due to small sample sizes, study differences in genotyping method or population substructure, or other factors. The general Chinese population occupies a vast country such that cultures and habits, such as personality, diet, living environment, and customs, can vary greatly among regions, for example, between southern and northern China. In this meta-analysis, four of the included studies were from northern China, and the remaining five were from southern China. Xu et al. showed that the greatest genetic differentiation of the Chinese Han population occurred between the northern Han Chinese and the southern Han Chinese[33]. In addition, Yang et al. reported marked geographical variation in the prevalence of *MTHFR* C677T, A1298C and MTRR A66G gene polymorphisms among different Chinese Han populations[34]. Differences among studies regarding the relationship between the *MTHFR* C667T polymorphism and male infertility risk may also be associated with variation in the nutritional status of people among different regions of China; for example, a higher vitamin intake can mask the biological effects of the *MTHFR* C667T polymorphism[35]. Regarding the *MTHFR* A1298C and *MS* A2576G polymorphisms, our results provided no evidence of either's association with male infertility risk in any genetic model, which is consistent with previous studies. Only three studies addressing the *MTHFR* A1298C and *MS* A2576G polymorphisms were included in the present meta-analysis; thus, studies with larger sample sizes are needed to further investigate the potential relationships of *MTHFR* A1298C and *MS* A2576G polymorphisms with male infertility risk.

Some limitations of the present study should be considered when interpreting the results. First, only nine studies were included in the meta-analysis, and their sample sizes were small; therefore, limited data were available. Second, we did not estimate the potential gene—gene and gene—environment interactions due to the lack of information available in the original studies. Third, other clinical data, such as sources of control, subject age, and semen quality, were not considered here due to a lack of information. Finally, although the funnel plot and Egger’s test indicated no remarkable publication bias, some publication bias may exist in the results because only published studies were retrieved.

## Conclusion

In summary, this meta-analysis provides evidence that the *MTHFR* C667T polymorphism may contribute to genetic susceptibility to the risk of male infertility in the Chinese population, whereas *MTHFR* A1298C and *MS* A2576G polymorphisms may have no impact. Nevertheless, large-scale, well-designed and population-based studies are needed to investigate the combined effects of these variants within the *MTHFR* gene or other folate-related enzyme genes in the Chinese population, which may lead to a comprehensive understanding of their potential roles in infertility.

## Supporting Information

S1 Checklist PRISMA 2009 Checklist. The PRISMA Checklist for our meta-analysis.
(DOC)

S2 Checklist Meta-analysis-on-genetic-association-studies. Meta-analysis on Genetic Association Studies Checklist.
(DOCX)

S1 File PRISMA 2009 flow diagram. The PRISMA 2009 flow diagram for our meta-analysis.
(DOC)
