# Supplementary material for: MTHFR C677T, A1298C and MS A2756G Gene Polymorphisms and Male Infertility Risk in a Chinese Population: A Meta-Analysis
Source: PLoS One. 2017 Jan 12;12(1):e0169789. doi: 10.1371/journal.pone.0169789 (PMC5230789; doi:10.1371/journal.pone.0169789)
Supplement: S1 File — The PRISMA 2009 flow diagram for our meta-analysis. (DOC) [file pone.0169789.s003.doc]

**
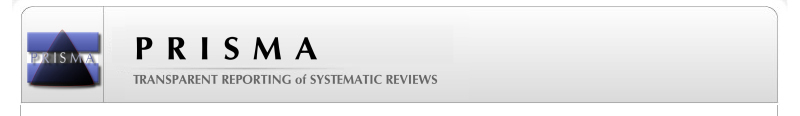
PRISMA 2009 Flow Diagram**

**Screening**

**Included**

**Eligibility**

**Identification**

Records remaining after duplicates removed
(n =140)

Records screened
(n = 140 )

Records identified through database searching
(n = 152)

Additional records identified through other sources
(n =0)

Records excluded:

Not reporting folate metabolism-related enzyme gene (n=69), Not reporting Chinese population (n=16), Not reporting male infertility (n=5), Reporting pathological mechanism (n=13), Animal study (n=6)

Full-text articles assessed for eligibility
(n =31 )

Full-text articles excluded:

Meta-analysis (n=6), Review article (n=15), Duplicate data (n=1)

Studies included in qualitative synthesis
(n = 9 )

**Included**

**Eligibility**

Full-text articles assessed for eligibility
(n =15 )

Full-text articles excluded,

1. Meta-analysis(n=3)
2. Review articles(n=2)
3. Data overlapped(n=1)

Studies included in qualitative synthesis
(n = 9 )

Studies included in quantitative synthesis (meta-analysis)
(n = 9 )

Studies included in quantitative synthesis (meta-analysis)
(n = 9 )
